# Antioxidant Properties and Enzyme Inhibitory Activities of *Eminium rauwolffii*: LC-MS/MS-Based Polyphenolic Profiling

**DOI:** 10.3390/plants15091311

**Published:** 2026-04-24

**Authors:** Kübra Aslan, Hasan Karageçili, Veysel Tahiroglu, Emrah Yerlikaya, Mustafa Abdullah Yılmaz, Mehmet Fidan, İlhami Gülçin

**Affiliations:** 1 Department of Chemistry, Faculty of Science, Ataturk University, 25240 Erzurum, Türkiye; kubra.aslan@atauni.edu.tr; 2 Department of Nursing, Faculty of Health Sciences, Siirt University, 56100 Siirt, Türkiye; 3 Department of Nursing, Faculty of Health Sciences, Sirnak University, 73100 Sirnak, Türkiye; veysel.tahiroglu@sirnak.edu.tr; 4 Department of Nutrition and Dietetics, Faculty of Health Sciences, Siirt University, 56100 Siirt, Türkiye; emrahyerlikaya@siirt.edu.tr; 5 Department of Analytical Chemistry, Faculty of Pharmacy, Dicle University, 21280 Diyarbakır, Türkiye; mustafaabdullahyilmaz@gmail.com; 6 Department of Biology, Faculty of Science and Arts, Siirt University, 56100 Siirt, Türkiye; mfidan7384@hotmail.com; 7 Rectorate of Agri Ibrahim Cecen University, 04100 Agri, Türkiye

**Keywords:** *Eminium rauwolffii*, antioxidant, acetylcholinesterase, α-glycosidase, carbonic anhydrase, phenolic compounds

## Abstract

*Eminium rauwolffii* (Blume) Schott var. *rauwolffii* is a member of the Araceae a large and mainly tropical family distributed worldwide. The Eminium species are utilized for various purposes including therapeutic uses in traditional medicine and as food. To analyze the antioxidant properties of water extract of *E. rauwolffii* (WEER) and ethanol extract of *E. rauwolffii* (EEER), 2,2’-azino-bis-3-ethylbenzthiazoline-6-sulphonic acid (ABTS^•+^) radical and 1,1-diphenyl-2-picrylhydrazyl (DPPH^.^) free radical scavenging, Fe^3+^-2,4,6-tris(2-pyridyl)-S-triazine (TPTZ) and Cu^2+^ reducing assays were studied. Antioxidant activities and reducing properties of both extracts were compared to standard antioxidants: BHT, BHA, α-Tocopherol, and Trolox. The IC_50_ values of EEER for radical scavenging were higher than those of standard antioxidants (25.35 ± 1.42 μg/mL for ABTS^•+^ and 106.80 ± 1.88 μg/mL for DPPH^•^). The total phenolic and flavonoid quantities in WEER and EEER were measured in the range of 189.78 ± 0.01 to 298.54 ± 0.01 mg GAE/g and 89.37 ± 0.01 to 178.95 ± 0.01 mg QE/g, respectively. The IC_50_ values for EEER and WEER against α-glycosidase, acetylcholinesterase (AChE), butyrylcholinesterase (BChE) and carbonic anhydrase I and II (hCA I and II) enzymes were 10.79 ± 5.61 to 13.18 ± 5.77, 36.14 ± 4.61 to 62.63 ± 1.67, 69.37 ± 7.36 to 37.48 ± 0.27, 81.30 ± 5.95 to 62.35 ± 8.03, and 29.34 ± 1.38 to 115.90 ± 3.3 µg/mL respectively. The antioxidant activity and enzymes inhibitory capacity of WEER were close, and comparable to the capacity demonstrated by the standards. The amount of sixteen compounds was identified from EEER. Numerous phytochemicals, including cynaroside, p-coumaric acid, cosmosiin, caffeic acid, and quinic acid, were quantitatively determined using the LC-MS/MS method. This clearly indicates that phenolic- and flavonoid-rich *E. rauwolffii* may have potential in the management of glaucoma, Alzheimer’s disease, diabetes, cardiovascular, and cancer disorders.

## 1. Introduction

*Eminium rauwolffii* (Blume) Schott var. *rauwolffii* is a member of the Araceae, a large family, also referred to as aroids. Over 8000 species and roughly 117 genera make up the broad, mostly tropical family Araceae. Aroids comprise 42 species and 9 genera in Turkey. In Turkey, there are six taxa that belong to the genus Eminium (Araceae) [1,2,3]. Eminium species are utilized for treating a variety of ailments, such as internal illnesses, diarrhea, abdominal pain, and gastrointestinal issues, according to ethnobotanical archives [4,5,6]. In addition to its anti-bacterial and antioxidant properties, Eminium taxa essential oils and extracts are utilized in traditional medicine. Eminium species have yielded luteolin and β-sitosterol [7,8]. Eminium taxa plant extracts and oils have been known to exhibit antioxidant and anti-bacterial properties. Aroids are therapeutic plants that are utilized in many cultures’ traditional medical practices. They can also be used as food [9]. These findings highlight the biological relevance of Eminium species.

Plant-derived bioactive compounds have attracted considerable attention due to their biological activities. Oxidative stress and the associated persistence of inflammation are the outcomes of an overabundance of free radical generation. Increased free radical levels and decreased antioxidant defense levels can result in oxidative stress [10]. Reactive oxygen species (ROS) and reactive nitrogen species (RNS) might harm proteins, lipids, and DNA in an organism’s cells and tissues, as well as promote cell death by inducing necrosis and apoptosis and breaking down the extracellular matrix [11]. Excessive ROS production results in oxidative stress in unhealthy situations [12]. In the processed food industry as well as other fields, natural chemicals derived from various plant sources have proved essential. Modern medications are mostly derived from natural sources. Consuming antioxidants found in natural compounds has been shown to improve human health and effectively lower the incidence of ROS and illnesses linked to oxidative stress [13]. It has been shown that several antioxidant compounds found in plant sources may scavenge free radicals or active oxygen better than synthetic antioxidants [14]. There is a growing demand from consumers for natural antioxidants, and efforts to investigate natural antioxidant sources have been spurred [15,16]. Thus far, a wide range of antioxidants found in plants has been identified [17]. The therapeutic herbs have a high polyphenol composition. The main natural antioxidants found in human diets come from fruits, grains, and plants [18]. Phytochemical substances offer protection against oxidative stress-induced and degenerative ailments [19]. Therefore, evaluating antioxidant capacity is important to understand the biological potential of plant extracts.

In addition to antioxidant properties, enzyme inhibition is another important mechanism associated with plant-derived compounds. Alzheimer’s disease (AD) is regarded as one of the most significant health concerns of contemporary times worldwide. Among various therapies, blocking AChE and BChE enzymes has been established for AD [20]. Neuropsychiatric symptoms and behavioral abnormalities are the major clinical manifestations of AD [21,22,23]. The progression of AD may be slowed by ingesting fruits and vegetables high in antioxidants [24,25]. Bioactive compounds were commonly employed as AChE inhibitors in clinical studies [26].

Based on the quantity of insulin released by the pancreatic beta cells and the biological action of the secreted insulin, diabetes is classified as type-1 diabetes mellitus (T1DM) or type-2 diabetes mellitus (T2DM) [27]. T2DM is the most prevalent endocrine condition worldwide [28]. α-Glycosidase converts oligosaccharides and polysaccharides into glucose and fructose. α-glycosidase inhibitors are essential for managing T2DM and hyperglycemia in humans [24].

Carbon dioxide is reversibly hydrated by carbonic anhydrases (CAs), which participate in gluconeogenesis, lipogenesis, and ureagenesis processes [29]. CA inhibition is a therapeutic treatment for cancer, glaucoma, and infection [30,31]. They keep fluid equilibrium, especially in the stomach, kidneys, and eyes. CA inhibitors can eliminate the increased intraocular pressure caused by glaucoma [32,33].

AChE is closely linked to AD, as it hydrolyzes acetylcholine, and its inhibition improves cognitive function. CA II plays a key role in regulating intraocular pressure. α-Glycosidases are digestive enzymes responsible for carbohydrate breakdown. Overall, dysregulation of these enzymes is associated with neurodegenerative disorders, metabolic diseases, and ocular conditions [22,27]. Thus, these enzymes are widely used as biochemical targets in inhibition studies.

The novelty of this study lies in providing the first comprehensive and integrated evaluation of *E. rauwolffii*, combining LC-MS/MS-based polyphenolic profiling, multiple antioxidant assays, and multienzyme inhibition studies targeting α-glycosidase, AChE, BChE, and CA enzymes. To the best of our knowledge, no previous study has simultaneously investigated all these biological activities together with detailed phytochemical characterization for this species. This approach enables a direct relationship between phytochemical content and biological activity to be established.

Inhibition research carried out on acetylcholinesterase (AChE) enzyme to determine the anti-Alzheimer’s disease effects of *E. rauwolffii* plant extracts. The association with glaucoma was determined by analyzing the inhibition of the CA II enzyme. The IC_50_ values for EEER and WEER were determined, and their antidiabetic potential on α-glycosidase was examined. The antioxidant capacity of *E. rauwolffii* was further assessed using FRAP, cupric ions reduction, DPPH and ABTS radicals scavenging assays, as well as determining the total quantities of phenolic and flavonoid compounds. The LC-MS/MS approach was used to analyze phenolic compounds. Accordingly, the aim of this study is to evaluate antioxidant properties, enzyme inhibitory activities, and phytochemical composition of *E. rauwolffii* extracts in a comprehensive manner.

## 2. Results

**Figure 1:**
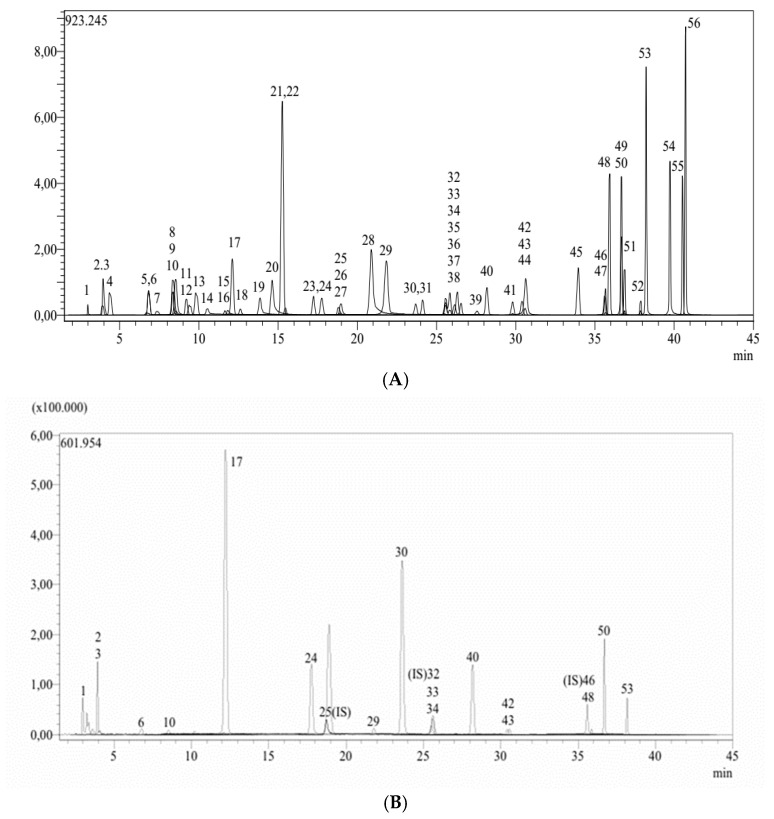

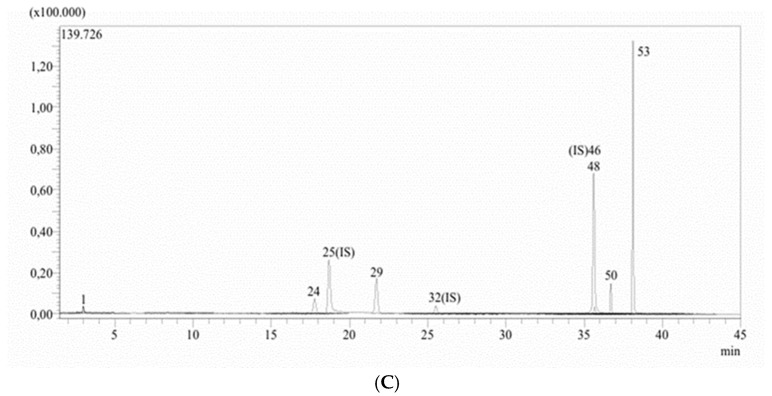
(**A**). Chromatogram of all standard phenolic compounds analyzed by the LC-MS/MS method. (**B**). LC-MS/MS Chromatogram of EEER (ethanol extract of *E. rauwolffii*). (**C**). LC-MS/MS Chromatogram of WEER (water extract of *E. rauwolffii*). 1. Quinic acid, 2. Fumaric acid, 3. Aconitic acid, 6. Protocatechuic acid, 10. Protocatechuic aldehyde, 17. Caffeic acid, 24. p-Coumaric acid, 29. Salicylic acid, 30. Cynaroside, 33. Rutin, 34. Isoquercitrin, 40. Cosmosiin, 42. Astragalin, 43. Nicotiflorin, 48. Naringenin, 50. Luteolin, and 53. Apigenin.

This study demonstrated a substantial number of flavonoids and phenolic compounds in *E. rauwolffii*. The total phenolic and flavonoid quantities in WEER and EEER were measured in the range of 189.78 ± 0.01 to 298.54 ± 0.01 mg GAE/g and 89.37 ± 0.01 to 178.95 ± 0.01 mg QE/g, respectively. The measurements are given in Table 1 and Figure 1. These abundant chemicals further have a direct impact on the plant extract’s antioxidant capacity. According to this research, *E. rauwolffii* has an equally high efficacious level of polyphenolics. Utilizing 53 phenolic molecules as references, the LC-MS/MS approach was employed to determine the main natural ingredients in *E. rauwolffii* extracts. By comparing their chromatographic properties, UV spectra, and MS data with reference compounds, the phenolic molecules were identified. The selection of standard compounds was based on their common occurrence in phenolic-rich plant extracts and their relevance in phytochemical profiling studies. These compounds were used as representative markers for the qualitative and quantitative evaluation of the extracts. A total of 17 secondary metabolites were identified in EEER, including 5 phenolic compounds in high quantity (quinic acid, fumaric acid, caffeic acid, p-coumaric acid, and protocatechuic acid) and three flavonoids (cynaroside, cosmosiin and luteolin). The major compounds identified in EEER are presented in Table 2.

**Figure 2:**
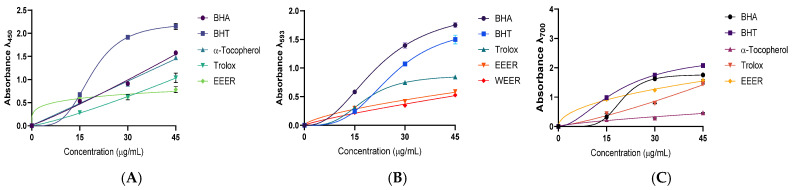
Reducing abilities of EEER (ethanol extract of *E. rauwolffii*) and WEER (water extract of *E. rauwolffii*) (**A**). Cupric ions (Cu^2+^) reducing ability, (**B**). Fe^3+^-TPTZ complex reducing ability and (**C**). Ferric ions (Fe^3+^) reducing abilities of EEER, WEER and standards. Fe^3+^ and Cu^2+^ reduction methods could not be determined despite several repetitions, so no results were found for EEER.

The reducing capacities of the extracts were determined using Cu^2+^, Fe^3+^, and FRAP assays, and the results are presented in Figure 2 and Table 3.

**Figure 3:**
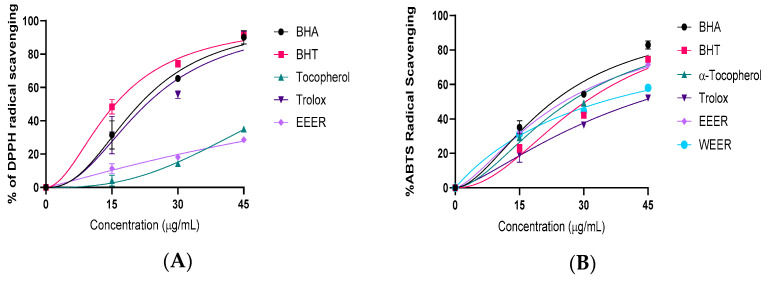
Radical scavenging effects of EEER (ethanol extract of *E. rauwolffii*) and WEER (water extract of *E. rauwolffii*) (**A**). DPPH free radical scavenging ability, (**B**). ABTS radical scavenging ability of EEER, WEER and standards.

The radical scavenging capacities of EEER and WEER were evaluated using DPPH and ABTS radical scavenging assays. The results are summarized in Figure 3 and Table 4.

**Figure 4:**
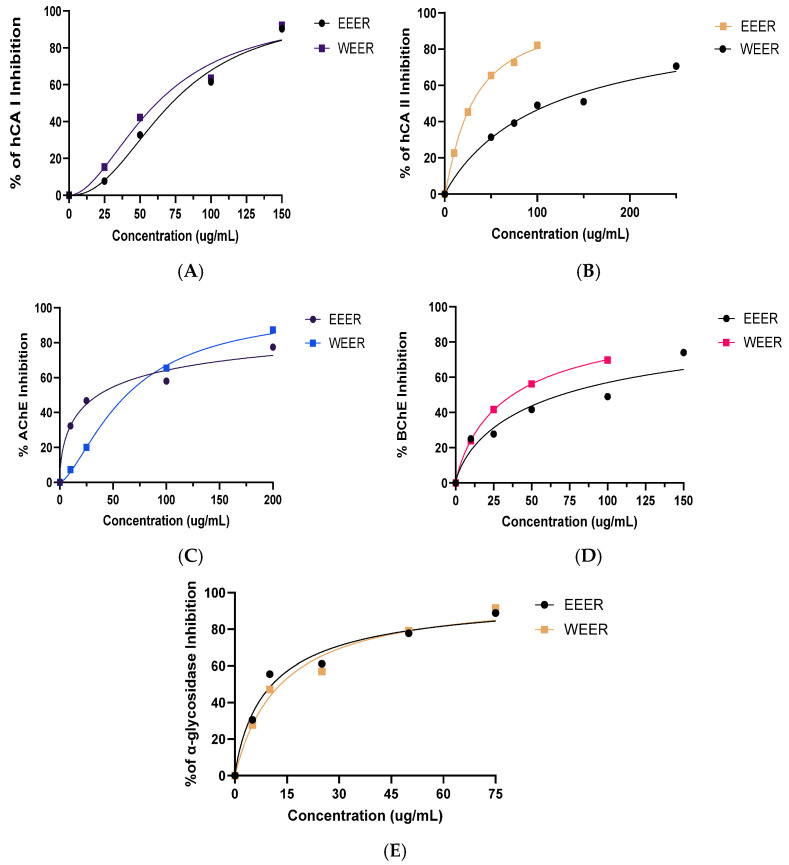
The half-maximal inhibitory concentration (IC_50_) graphs of *E. rauwolffii* extracts against human carbonic anhydrase I and II (hCAs I and II) (**A**,**B**), acetylcholinesterase (AChE) (**C**), butyrlcholinesterase (BChE) (**D**), and α-glycosidase enzyme (**E**).

The enzyme inhibitory activities of EEER and WEER against α-glycosidase, AChE, BChE, and both CA isoenzymes are presented in Table 5. EEER exhibited stronger inhibitory activity compared to WEER for most of the tested enzymes (Figure 4).

## 3. Discussion

Natural phytochemicals have been implicated in the treatment of many illnesses, such as diabetes, cancer, heart disease, and aging, according to previous studies [34]. It has also been reported that medicinal and aromatic plants with higher antioxidant activity contain a wide range of phenolic compounds. Consequently, these plants may include antioxidants that might aid in the fight against cancer and other diseases [35]. Plants provide phenolic secondary compounds, which are an important and necessary component of human nutrition. They are given given a lot of attention because of the antioxidant qualities of their bioorganic activity [36]. The *E. spiculatum* extracts results showed high content of phenolic contents (107.3 ± 3.1 and 99.7 ± 2.0 mg GAE/g for 50 and 100% ethanolic extracts, respectively) and flavonoid content (67.9 ± 2.9 and 45.6 ± 2.8 mg catechin/g for 50 and 100% ethanolic extracts, respectively) [37]. Total phenolic and flavonoid concentrations were most crucial in extracts from the flowers and leaves of *E. intortum* (50.82 ± 0.71 mg GAE/g and 65.08 ± 0.38 RE/g, subsequently) [3]. Total phenolic concentration ranged from 21.65 to 28.85 mg GAE/g, according to research on *A. elongatum*. The greatest overall phenol value was found in the methanol/water extract. Methanol extract has the greatest total flavonoid concentration (36.77 mg RE/g) [6]. Extraction conditions were not kept identical for water and ethanol extracts, as the procedures were optimized according to the physicochemical properties and extraction kinetics of each solvent. It is well established that different solvents require different extraction conditions to achieve efficient recovery of phytochemicals, due to variations in polarity, boiling point, and mass transfer characteristics [15]. Therefore, solvent-specific extraction protocols were applied in this study rather than under strictly identical conditions.

The LC-MS/MS analysis provided a comprehensive quantitative characterization of the phytochemical composition of both EEER and WEER of *E. rauwolffii*, revealing notable differences in their chemical profiles. Overall, the EEER extract was found to contain a higher diversity and concentration of phenolic compounds compared to WEER, which can be attributed to the greater extraction efficiency of ethanol for semi-polar phytochemicals such as flavonoids and phenolic acids. In contrast, the WEER extract primarily contained more polar constituents and generally exhibited lower concentrations of several key phenolics. Among the identified compounds, quinic acid, caffeic acid, and p-coumaric acid were detected as major components, although their concentrations differed between the two extracts. These phenolic acids were more abundant in the EEER extract, suggesting that ethanol is more effective in extracting compounds with moderate polarity. Similarly, flavonoid derivatives such as cosmosiin (apigenin-7-O-glucoside) were predominantly enriched in EEER, further supporting the enhanced extraction capacity of ethanol for bioactive flavonoids. On the other hand, the WEER extract showed a comparatively limited phytochemical profile, which may explain its generally lower biological activity observed in several assays. The observed differences between WEER and EEER can be attributed not only to solvent polarity but also to solvent-specific extraction conditions. It is well established that extraction parameters such as temperature and duration significantly influence the recovery of phytochemicals. Therefore, the results presented in this study reflect the combined effects of solvent polarity and optimized extraction conditions, rather than a direct comparison under identical extraction parameters [16].

Major phenolic compounds such as caffeic acid, p-coumaric acid, quinic acid, and flavonoids (e.g., cosmos and luteolin) are known to exert antioxidant effects through multiple mechanisms, including hydrogen atom transfer (HAT), single electron transfer (SET), metal ion chelation, and modulation of redox-sensitive pathways [12,16,32].

These compositional differences are in strong agreement with the biological activity results. The higher total phenolic and flavonoid content of EEER correlates well with its superior antioxidant capacity in DPPH, ABTS, and reducing power assays, as well as its stronger enzyme inhibition effects. Phenolic acids such as caffeic acid and p-coumaric acid are well known for their radical scavenging ability [12] and inhibitory effects on enzymes like α-glycosidase and acetylcholinesterase, while flavonoids contribute through multiple mechanisms, including metal chelation and interaction with enzyme active sites. Therefore, the enhanced biological activities of EEER can be largely attributed to its richer and more diverse phenolic compositions. In contrast, the lower activity observed in WEER may be explained by both the reduced concentration and the narrower range of bioactive compounds. Water extraction, although representative of traditional usage, may not efficiently recover fewer polar phytochemicals that significantly contribute to antioxidant and enzyme inhibitory activities. Nevertheless, the presence of certain phenolic acids in WEER still indicates that it retains some degree of biological potential.

Overall, the LC-MS/MS results clearly demonstrate that solvent polarity plays a critical role in determining the phytochemical composition and, consequently, the biological activity of *E. rauwolffii* extracts. Compounds with similar structures and close retention times, such as rutin and isoquercitrin, may partially overlap or produce related signals due to in-source fragmentation; therefore, results are interpreted within the limitations of the targeted method. The integration of phytochemical profiling with bioactivity data highlights that the superior pharmacological potential of EEER is closely linked to its higher content of phenolic acids and flavonoids, emphasizing the importance of the extraction strategy in maximizing the therapeutic value of plant-derived products.

According to reports, the antioxidant, antimicrobial, and in vitro suppression of diabetes enzymes exhibited by chemicals detected by LC-MS/MS in a variety of plant extracts, including cynaroside and cosmosiin may have antidiabetic properties [38]. A study’s findings further demonstrate that p-coumaric acid is an effective antioxidant that enhances the activity of the antioxidant enzymes catalase, Glutathione-*S*-transferase, and superoxide dismutase, as well as the lipid peroxidation changes brought on by diabetes [39]. Fumaric acid esters’ anti-inflammatory and antioxidant properties in neurodegenerative illnesses were shown in a prior study [40]. Coffee, fruits, and vegetables contain caffeic acids, one of the most common plant-based polyphenols. These acids have two phenolic hydroxyl moieties. Numerous phenolic compounds including caffeic acid, which has been linked to anti-cholinesterase in tissues or animal models, as well as in cell line research, and have a neuroprotective effect in behavioral studies [41]. By generating apoptosis-mediated cytotoxicity in breast cancer cells, quinic acid has been shown to possess anticancer effects [42].

One of the most important markers of a molecule’s possible antioxidant activity may be its reduction capability. By receiving electron donations from antioxidant compounds, reactive radicals can be transformed into more stable and non-reactive species [43]. The reduction potentials of phenolic combinations extracted from *E. rauwolffii* were evaluated using three different reduction systems, including the reducing abilities of CUPRAC, Fe^3+^ and Fe^3+^-TPTZ. DPPH and ABTS radical scavenging assays were used to assess the radical scavenging hallmark of *E. rauwolffii*, which contains natural chemicals that may have reducing qualities, neutralizing ROS and oxidants.

The cuprous neocuproine chelate [Cu(I)-Nc] is reduced in the presence of antioxidants to the chromogenic oxidant neocuproine (bis(2,9-dimethyl-1,10-phenanthroline), which exhibits maximum light absorption at 450 nm, in the CUPRAC method of total antioxidant capacity (TAC) assay [44]. For a range of antioxidants, the Cuprak antioxidant technique is quick, stable, affordable, selective, and easy to use [45,46].

The overall reduction capacity of plant extracts or pure antioxidant compounds can be determined using the FRAP assay. The FRAP test is based on an electron transfer mechanism that uses ferric salt as an oxidant. Fe^2+^’s colorful association with TPTZ, which has a maximum absorbance at 593 nm, allows for spectrophotometric detection [47]. This approach works very well for determining the reduction capacities of bioactive compounds. The FRAP assay uses the sample’s antioxidants as reductants in a redox-linked colorimetric reaction first. Furthermore, it is simple to standardize the FRAP assay procedure, and it is simple to follow [48]. The FRAP test was developed to assess the reduction of ferric iron in aqueous solutions of pure compounds and biological fluids. Furthermore, it has been used to evaluate the antioxidant potential of polyphenols [49]. In this investigation, the yellow hue of the test suspension changes to a range of green and bluecolors, indicating the strength of a reducing agent or antioxidant sample.

*E. rauwolffii* species extracts were tested for their capacity to reduce Fe^3+^ by direct reduction of Fe^3+^(CN^−^)_6_ to Fe^2+^(CN^−^)_6_ and the absorbance that arises from the formation of the Perl’s Prussian Blue complex upon the addition of excess ferric ions (Fe^3+^). To evaluate the reducing ability of extracts from Eminium species, the ferric reducing technique of Oyaizu [50] was used with a slight change [51]. In this method, Fe^3+^ would be changed into Fe^2+^ in the presence of Eminium plant extracts or reductants. The addition of ferric ions (Fe^3+^) to Eminium species leads to the formation of Fe_4_[Fe(CN^−^)_6_]_3_ complex, resulting in a maximum absorption at 700 nm [52,53]. The higher the polyphenolic content in the ethanol extract of *E. rauwolffii*, the higher the reducing activities of metal ions and the higher antioxidant activities. The higher content of phenolics found in this study statistically gave higher antioxidant and enzyme inhibition results.

Radical scavenging-based spectrophotometric techniques are consistently used to evaluate the antioxidant quality of substances, drinks, meals, and herbal extracts. The DPPH∙ and ABTS^∙+^ scavenging methods are also highly sensitive, simple to use, fast, selective, and reproducible. As a result, the ability to eliminate radicals is often described using them [54]. Antioxidant capacity is frequently analyzed using the DPPH∙ method. This method involves converting DPPH∙ to DPPH-H, which is not a radical [55,56]. A newly produced DPPH solution displays a rich purple color with a peak of absorption at 517 nm. This purple hue frequently disappears when the medium contains an antioxidant. The drop in absorbance is an indicator of the free DPPH due to the antioxidant’s effect [57]. *E. rauwolffii* is regarded to have medicinal antioxidant capabilities if it has the capacity to scavenge DPPH∙. The DPPH∙ scavenging capacity of EEER and WEER was determined, and an IC_50_ value was calculated (Table 2). Radical scavenging of *E. rauwolffii* was dependent upon the concentration. Standard radical scavenger compounds, including Trolox, α-tocopherol, BHT, and BHA, as well as *E. rauwolffii* IC_50_ values, were 106.80 µg/mL for EEER, 15.77 µg/mL for BHT, 21.33 µg/mL for BHA, 22.88 µg/mL for Trolox, and 56.88 µg/mL for α-tocopherol. Correlated to normal antioxidants and the findings of other investigations, EEER most likely has a slightly lower and -mild antioxidant capacity, as demonstrated in this investigation.

The extracts of *E. spiculatum* species exhibited notable antioxidant scavenging capacity against the stable radical DPPH, with values of 298.7 ± 3.3 and 190.8 ± 2.2 µmol Trolox/g for 50 and 100% ethanolic extracts, respectively [37]. A study conducted on *A. elongatum* Steven, another commonly used Kari species, showed that the antioxidant activity of the extracts ranged from 24.36 to 133.08 mg TE/g in the ABTS assay and from 1.79 to 38.90 mg Trolox equivalents per gram in the DPPH assay [6]. The total antioxidant capacity by DPPH assay was 340 ± 32 and 402 ± 20 mg TE/10 for *A. elongatum* taken from two neighboring cities, Gümüshane (C1) and Giresun (C2), respectively. However, the CUPRAC assay results were 1619 ± 99 and 1839 ± 63 mg TE/100 g for C1 and C2 infusions, respectively [7]. Total antioxidant activity and reducing capacity vary depending on the elevation of the geographical region where the plants are harvested, climate, environmental stress, time of harvest, water, salinity, soil type, sunlight and the strength of phenolic and flavonoid compounds, which are secondary metabolites.

The ABTS radical scavenging capacity of extract and references recorded, Trolox, α-tocopherol, BHT, and BHA, as well as *E. rauwolffii* IC_50_ values, were provided in the following ranges: 25.35 μg/mL for EEER, 34.42 μg/mL for WEER, 30.43 μg/mL for BHT, 22.85 μg/mL for BHA, 43.08 μg/mL for Trolox, and 26.87 μg/mL for α-tocopherol (Table 2) The results indicated that EEER and WEER had strong ABTS^•+^ scavenging activity when compared to standards. The radical scavenging effect of the EEER sample was greater than that of practically all reference antioxidants, except for BHA.

The ABTS radical scavenging properties of the extract and standards in a different investigation with *A. elongatum* were as follows: distilled water with *A. elongatum* (96.63%), BHA (95.84%) > BHT (93.25%) > *A. elongatum* (ethanol) (94.15%) [58]. No3 scavenging of radicals (32.20 ± 1.26 and 54.34 ± 0.53 mg TE/g for DPPH and ABTS, respectively) was observed in leaf extracts of *E. intortum* [3]. Eminium and Arum-related species exhibit similar antioxidant activity. Various species of the Arum family occur naturally in the wild and have not yet been widely cultivated. Their safety requires the appropriate knowledge and experience. These species contain phenolic and flavonoid compounds that may play a role in plant survival while also representing potential value for human nutrition and pharmacological applications.

In particular, the antioxidant capacity (DPPH, ABTS, and reducing power assays) and total phenolic/flavonoid contents of *Eminium rauwolffii* were compared with those reported for related Araceae species such as *Arum elongatum* and *Eminium intortum*, which are known to exhibit strong antioxidant activities associated with high phenolic content [3,6,7]. These studies similarly reported that phenolic acids and flavonoids are the primary contributors to antioxidant capacity.

α-Glycosidase is associated with diabetes, cancer, and viral infections and is essential for the metabolism of carbohydrates. Given its many biological roles, the biological enzyme α-Glycosidase is considered a prospective therapeutic target. Inhibitors of α-glycosidase, have been extensively investigated such as Acarbose and Miglitol, competitively inhibit the breakdown of carbohydrates, inhibit the brush boundary α-glycosidase in the small intestine, and reduce the hyperglycemia [59,60]. Inhibitors of α-glycosidase can be a crucial part of the treatment plan for T2DM. Postprandial hyperglycemia is a significant and early consequence of diabetes that can be linked to secondary illnesses connected to diabetes, and controlling blood glucose levels can prevent the advancement of these disorders [61]. In vitro α-glycosidase enzyme inhibition in our lab investigation illustrated the mechanism of ameliorating effects of extracts of *E. Rauwolffii* species on human diabetes.

AChE inhibitors are used to treat Alzheimer’s disease (AD). Nevertheless, there are a lot of negative side effects from such medications. Therefore, there is an urgent need for new, potent antioxidants and AChE agents [62]. The highest AChE inhibitory effects were likewise found to be exhibited by aromatic compounds and, to a lesser extent, aliphatic molecules [63]. Although acetylcholinesterase inhibitors are employed to treat Alzheimer’s disease, they merely offer short-term alleviation. Medicinal herbs typically contain large amounts of cholinesterase inhibitors. Phenolic compounds are principally accountable for the inhibition of cholinergic enzymes in medicinal plants [64]. The highest anticholinesterase activity was observed in *E. intortum* flowers (2.72 ± 0.03 mg GALAE/g) [3]. However, AChE and BChE enzyme inhibition using *E. rauwolffii* gave lower inhibition effects than the reference inhibitor. In this study, it was shown in Table 3 that *E. rauwolffii* ethanol and water extract inhibited the AChE and BChE enzymes efficiently. This study is limited to in vitro biochemical assays, which do not fully reflect the complex biological systems involved in AD. Therefore, further investigations, including cellular and in vivo studies, are required to establish a definitive link between antioxidant properties and therapeutic outcomes.

The physiologically dominant cytosolic isoforms hCA I and hCA II are broadly distributed and implicated in several diseases such as glaucoma, altitude sickness, edema, and epilepsy [65]. Ocular pressure is decreased by decreased aqueous humor secretion and HCO_3_-production brought on by CA II suppression [66]. The deterioration of the optical nerve, which is mostly associated with high intraocular pressure (IOP), is the hallmark of glaucoma, a multifactorial optic nerve disease that can cause blindness. Because hCA inhibitor medications such as acetazolamide, brinzolamide, and dorzolamide effectively lower IOP after topical treatment, novel therapeutic considerations are required [67]. *E. rauwollfii* herbs have a high potential to be cultivated and used for a natural and healthy life with their antioxidant, metal reducing capacity and inhibition of metabolic enzymes that are efficient in the prophylaxis of diabetes, glaucoma and Alzheimer’s ailments, which were examined and determined in this research. Moreover, in this work, the inhibition of CA I and CA II enzymes by plant extracts from *E. rauwolffii* has been added to the literature for the first time. The study’s findings indicate that when the solution of the plant is appropriately adjusted, *E. rauwolffii* extracts may be helpful in the treatment of glaucoma.

Furthermore, the observed enzyme inhibition activities (α-glycosidase, AChE, and CA) were discussed in relation to other plant-derived extracts rich in compounds such as caffeic acid, p-coumaric acid, and flavonoids, which have been widely reported to exhibit inhibitory effects on these enzymes [19,52]. The comparable IC_50_ values obtained in our study suggest that *E. rauwolffii* demonstrates a bioactivity profile consistent with other medicinal plants containing similar phytochemical constituents.

## 4. Materials and Methods

### 4.1. Chemicals

Acetylcholinesterase and α-glycosidase enzymes; acetylcholine iodide and p-nitrophenyl-D-glucopyranoside substrates; DPPH, ABTS, and neocuproine (2,9-dimethyl-1,10-phenanthroline); synthetic reference antioxidants; BHT, BHA, Trolox and α-tocopherol; nitroblue tetrazolium (NBT); Ferrozine (3-(2-pyridyl)-5,6-bis(4-phenyl-sulfonic acid)-1,2,4-triazine); trichloroacetic acid chemicals (TCA); and standard phenolic compounds of LC-MS/MS were purchased from Sigma (Sigma-Aldrich GmbH, Sternheim, Germany). Ammonium thiocyanate is purchased from Merck. Accordingly, the remaining ingredients were bought from Merck (Darmstadt, Germany) or Sigma-Aldrich (Sigma-Aldrich GmbH, Sternheim, Germany).

### 4.2. Plant Material

All references to *E. rauwolffii* in the manuscript correspond to *Eminium rauwolffii* (Blume) Schott var. *rauwolffii*, which was collected from Şırnak province (Cizre Güçlükonak Road, Dirsekli, 73200 Ulaş, Şırnak Center), at an altitude of 1100–1600 m. *E. rauwolffii* was recorded in the Siirt University Herbarium (Herbarium number: SUFAF 1731) by plant taxonomist Mehmet Fidan, who assigned the species the number SUFAF 1731. The exact GPS coordinates of the collection site are 37°23′15.9″ N, 42°07′48.3″ E. Due to the potential inhibitory effects of ethanol as a solvent, the ethanol extract of *E. rauwolffii* (EEER) was dissolved in a minimal volume of DMSO for enzyme inhibition experiments and diluted with water to the desired concentration.

### 4.3. Preparation of the Ethanol Extract of E. rauwolffii Species

**Figure 5:**
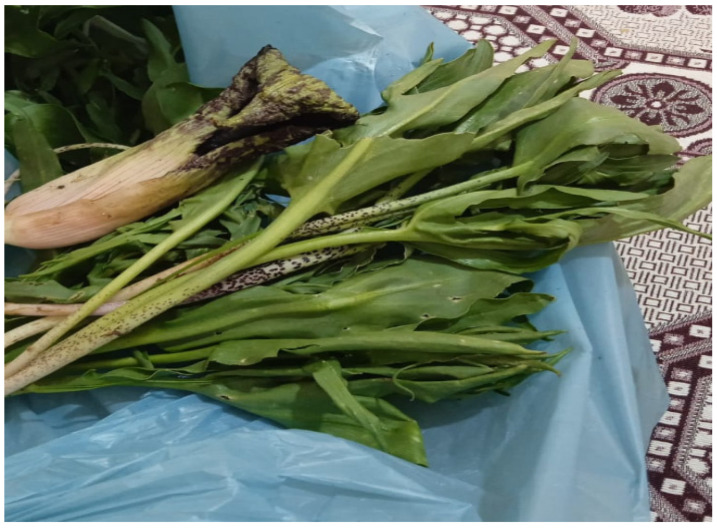
*E. rauwolffii* is an edible plant in Şırnak and Siirt provinces.

The extraction procedure was performed as previously indicated [15]. A mill was used to grind 25 g of dried aerial parts of the *E. rauwolffii* plant (Figure 5), and 100 mL of distilled and deionized water was used to make aqueous extracts of the samples. This mixture was cooked for twenty minutes using a magnetic stirrer (Heidolph Instruments GmbH & Co. KG, Schwabach, Germany). The extracts of the filter were lyophilized (Labconco Lyophilizator, Freezone, Kansas City, MO, USA) at a room temperature of −50 °C and 5 mmHg of pressure [68]. The specimen’s ethanol extracts were made by crushing 25 g of arid *E. rauwolffii* plant, mixing it with 100 mL of ethanol, and swirling it for an hour on a magnetic stirrer. The extracts were filtered, and then the filtrates were gathered. To get rid of the ethanol, the temperature was programmed at 50 °C in a rotary evaporator. All of the plant extracts were kept at a temperature of 20 °C before being employed in the experiments [69].

### 4.4. Total Phenolic Contents

The total phenolic content was determined according to previous study [70], which developed by Singleton and Rossi [71]. It was utilized to analyze the phenolic content of EEER and WEER by implementing small adjustments to the approach [15]. To the 1.0 mL of Folin–Ciocalteu reagent, 0.5 mL of each extract specimen was added. Then, 0.5 mL of carbonate (1%) was added to the mixture to neutralize it and combine it well. Following a two-hour dark incubation period at room temperature, the absorbance was measured at 760 nm towards a blank sample composed of distilled water. The linear regression equation of the calibration curve for gallic acid was applied to determine the phenolic quantities. Milligrams of gallic acid equivalents (mgGAE) per gram of EEER or WEER are the unit of measurement used to express the values of the phenolic content analysis.

### 4.5. Determination of the Total Flavonoid Contents

The total flavonoid levels in EEER and WEER are calculated using a colorimetric test outlined by Gulcin et al. [72]. The EEER and WEER samples were mixed with 0.5 mL of 95% methanol. Following the supplements of 1.5 mL of 10% Al(NO_3_)_3_, 0.5 mL of CH_3_COOK (1.0 M), and 2.3 mL of distilled water, the samples were vortexed and held in the dark and allowed to come to room temperature for forty minutes. The absorbance measurements are recorded at 415 nm [73]. Distilled water served as a blank and control. Quercetin’s flavonoid content was estimated using the calibration curve’s linear regression equation. Quercetin equivalents (QE), which are the results, are expressed as mg per gram of EEER or WEER.

### 4.6. LC-MS/MS Analysis

Quantitative analysis of phytochemicals was performed using a validated LC-ESI-MS/MS method based on multiple reaction monitoring (MRM) mode, as previously described by Yılmaz [74]. In this approach, compound analysis was achieved by combining retention time data with specific precursor ion (*m*/*z*) and corresponding product ion transitions, providing two orthogonal determination parameters. Each analyte was selectively monitored using optimized MRM transitions under defined collision energies, ensuring high sensitivity and specificity. Therefore, metabolite analysis was not based solely on retention time but also supported by MS/MS fragmentation patterns.

A Shimadzu Nexera UHPLC system coupled with a Shimadzu LCMS-8040 triple quadrupole mass spectrometer (Shimadzu, Kyoto, Japan) was used for quantitative evaluation of 53 phytochemicals. Chromatographic separation was performed on an Agilent Poroshell 120 EC-C18 column (150 mm × 2.1 mm, 2.7 µm; Agilent, Santa Clara, CA, USA) at 40 °C. The mobile phase consisted of (A) water containing 5 mM ammonium formate and 0.1% formic acid and (B) methanol containing 5 mM ammonium format and 0.1% formic acid. The gradient program was as follows: 20–100% B (0–25 min), 100% B (25–35 min), and 20% B (35–45 min). The flow rate and injection volume were 0.5 mL/min and 5 µL, respectively.

Mass spectrometric detection was carried out using an electrospray ionization (ESI) source operating in both positive and negative modes. Data acquisition was performed in MRM mode, where specific precursor-to-product ion transitions were monitored for each compound. The MS parameters were set as follows: drying gas (N_2_) 15 L/min, nebulizing gas (N_2_) 3 L/min, DL temperature 250 °C, heat block temperature 400 °C, and interface temperature 350 °C. Collision energies were optimized for each analyte to obtain maximum sensitivity and reproducible fragmentation patterns.

#### 4.6.1. LC-MS/MS Test Solution Preparation

The analytical methodology employed in this research was carried out in accordance with the most recent findings. An LC-MS/MS investigation was conducted at the Dicle University Central Research Laboratory. Yılmaz [74] created the method utilized in this research and modified it for *E. rauwolffii*. They were employed to investigate the phytochemical components in each EEER and WEER.

#### 4.6.2. Sample Preparation

In a volumetric flask, 100 mg of each of EEER and WEER were dissolved in 5 mL of ethanol–water (50:50, *v*/*v*). One milliliter of this mixture was subsequently transferred into another 5 mL volumetric flask and diluted to volume with ethanol–water (50:50, *v*/*v*). Following dilution, internal standards (Ferulic acid D3, rutin D3, and quercetin D3) were added to the solutions at concentrations of 20 mg/L, 1 mg/L, and 5 mg/L, respectively. The resulting solutions were then filtered through a 0.2 μm syringe filter prior to LC–MS/MS analysis.

A 1.5 mL aliquot of the final mixture was transferred into an autosampler vial, and 10 μL of the sample was injected into the LC–MS/MS system. The specimens in the autosampler were maintained at 15 °C [74]. The selection of compounds was guided by the scope of the validated LC-MS/MS method developed by Yılmaz [74], which includes a panel of standard compounds frequently used as reference markers in phytochemical analyses. Detailed validation parameters and MRM transition data are provided in the Supporting Information (Table S1), based on the validated method described by Yılmaz [74]. Although no additional quality control (QC) samples were specifically prepared for this study, the method was applied under the same validated analytical conditions, and system suitability was monitored through retention time consistency and signal stability.

### 4.7. Reducing Ability Assays

According to the techniques of Apak et al. [75], with little changes as defined, the Cu^2+^ reducing effects of each EEER and WEER were recorded [76]. To accomplish this, the same volumes of 250 µL of the EEER and WEER solution (10–30 µg/mL) in a glass test tube were added to the same quantities of the neocuproine mixture (7.5 mM), acetate buffer (0.25 mL, 1.0 M), and CuCl_2_ solution (10 mM). Using distilled water and stirring thoroughly, the total mixture volumes are adjusted to 2 mL. After sealing, the glass tubes are stored at 25 °C until they are used in investigations. After 30 min, their absorbances at 450 nm are finally recorded using a spectrophotometer (Shimadzu UV-1800 UV) [76]. The FRAP reducing power is based on the Fe^3+^-TPTZ reduction in acidic solution medium. At 593 nm, the reduced form of Fe^2+^-TPTZ is detected spectrophotometrically. The TPTZ solution (10 mM, 2.25 mL) and FeCl_3_ (20 mM, 2.25 mL) in buffer (2.5 mL, pH: 3.6, 0.3 M) were both present in the FRAP reagent solution. Following the addition of a 0.2 mL aliquot of the EEER and WEER to 1.8 mL of FRAP reagent, the absorbance is measured at 593 nm. A solution of phosphate buffer is used to create a blank sample [77]. A volume of 1.25 mL of phosphate buffer fluid (0.2 M, pH 6.6) and 1% (*w*/*w*) potassium ferrocyanide were combined with various concentrates of the EEER and WEER (0.75 mL in distilled water). The blend was then acidified using 1.25 mL of 10% *w*/*w* trichloroacetic acid and incubated for 30 min at 50 °C. After adding 0.25 mL of FeCl_3_ (0.1%) to create the blue complex, each sample’s absorbance was computed at 700 nm using a spectrophotometer (UV-1200, Shimadzu Corporation, Kyoto, Japan) [78].

### 4.8. Radical Scavenging Activities

#### 4.8.1. DPPH^•^ Scavenging Activity

According to the method of Blois [79] and had been utilized previously [80]. The radical removing capacity of EEER and WEER was measured. The day before the measurement, the DPPH solution was made. The mixture in the flask was covered with aluminum foil and left in darkness at 4 °C for 16 h while being stirred. Shortly after making a 0.1 mM DPPH suspension in ethanol, 2 mL of the EEER and WEER in ethanol at different concentrations (10–30 g/mL) were mixed with 0.5 mL of this solution. The sample of *E. rauwolffii* was vortexed and then incubated for 30 min at 30 °C in the dark. Absorbance was evaluated at 517 nm relative to blank samples [12].

#### 4.8.2. ABTS^•+^ Scavenging Activity

After adding 2 mM 2.45 mM potassium persulfate (K_2_S_2_O_8_) to ABTS in water to create ABTS^•+^, the mixture was left to rest for six hours at room temperature and in the absence of light. It took more than six hours for the absorbance to reach its peak and stabilize, even though the ABTS started to oxidize immediately. Before equilibrating the solution at 30 °C, the temperature at which all experiments are conducted. It is prepared for the experiment by diluting it in phosphate buffer (pH 7.4) to provide an absorbance at 734 nm of 0.7000 ± 0.02 in a 1 cm cuvette. Then, 1 mL of the ABTS^•+^ solution is mixed with 3 mL of EEER and WEER solutions in ethanol at 10–30 µg/mL. Following 30 min of mixing, the absorbance is measured, and the rate at which radicals are scavenged for each concentration is computed in comparison to a blank that contains no scavenger [80,81,82]. The decrease in absorbance indicates the level of decolorization.

### 4.9. Enzyme Inhibition Studies

#### 4.9.1. AChE Inhibition Study

EEER and WEER solutions’ inhibitory capabilities on AChE were determined using Ellman’s technique [83] as reported in a recent research [84]. Electric eels’ AChE serum was used to accomplish this. In conclusion, the enzyme blend (50 μL, 5.32 × 10^−3^ EU) was mixed with EEER and WEER concentration (10–30 μg/mL) in buffer (1.0 M Tris/HCl, 100 μL, pH 8.0). The reagents were maintained at 20 °C for 10 min. Subsequently, 50 µL of solutions including 5,5′-dithio-bis(2-nitro-benzoic acid (DTNB) (0.5 mM) and acetylthiocholine iodide (AChI) were added. The reaction was then initiated, and the absorbances of the combination were determined spectrophotometrically at 412 nm [85].

#### 4.9.2. α-Glycosidase Inhibition Study

The inhibiting effects of the EEER and WEER on α-glycosidase enzyme were investigated using the technique of Tao et al. [86], as elaborately explained [87]. Different quantities of extracts were put into phosphate buffer (75 μL, pH 7.4) for this reason. Then, 20 μL of α-glycosidase solution was added to the same buffer and incubated for 10 min. A 50 μL aliquot of p-nitrophenyl-D-glycopyranoside (p-NPG) dispersed in the same buffer was added to the resultant combination. The combination was incubated again at room temperature (37 °C) [88]. The absorbances at 405 nm were estimated using a phosphate buffer blend as a blank.

#### 4.9.3. CA Isoenzymes Inhibition Study

As previously reported, sepharose-4BL-tyrosine sulfanilamide affinity chromatography was utilized to extract and isolate both CA isoenzymes from human blood samples [89]. The Bradford method [89] was employed to measure the protein levels at 595 nm following the purification of the enzymes. The approach of Verpoorte et al. [90] was utilized to perform esterase activity assay, at 348 nm using a spectrophotometer (Shimadzu, UVmini-1240 UV–VIS) [91]. As a reference standard, acetazolamide (AZA) was utilized [92].

#### 4.9.4. Statistical Analysis

All measurements were performed in triplicate using the same extract (technical replicates). The results are given as mean ± SD. In the two-way ANOVA, significant differences were considered to have a value of *p* < 0.05. All data were processed, and graphs were created using GraphPad Prism 8.0.2. IC_50_ value refers in this study to the concentration of an inhibitor needed to inhibit an enzyme activity response by 50%. The calculation of the IC_50_ values was performed using the GraphPad Prism 8.4.0 non-linear regression-[inhibitor]-normalized response (y values 100 down to 0) model.

## 5. Conclusions

*Eminium rauwolffii* (Blume) Schott var. rauwolffii is commonly known as “kardi,” “kari,” and “wolf’s ear.” Eminium species have traditionally been used as edible plants in the regions where they grow and are commonly consumed as food, including in soups. Determination of its content by using phenolic and flavonoid profile employed LC-MS/MS and comparative analysis of antioxidant content (DPPH and ABTS), Cu^2+^, Fe^3+^ and FRAP reducing capacities with standard antioxidants. Additionally, this study represents the first comprehensive investigation of the inhibitory effects on α-glycosidase, AChE, and CA II enzymes, considering that *E. rauwolffii* is commonly consumed as a vegetable in omelets, stews, and soups. They are quite good at inhibiting enzymes, despite having less reducing and antioxidant properties than synthetic antioxidants. Both on their own and in the creation of functional foods, *E. rauwolffii* plant extracts can be utilized. *E. rauwolffii* has been found to have strong anti-glaucoma, anti-Alzheimer, antidiabetic, and antioxidant properties.

Extracts from *E. rauwolffii* have a measurable quantity of phenolic compounds and flavonoids, which provide them with antioxidant, antidiabetic and antiglaucoma features. According to the LC-MS/MS, the major components recognized in *E. rauwolffii* were cynaroside, p-Coumaric acid, cosmosiin, caffeic acid, and quinic acid. Furthermore, *E. rauwolffii* contains flavonoids and phenols, which may efficiently inhibit the enzymes hCA II, α-glycosidase, and AChE. Natural products made from *E. rauwolffii* can be utilized to treat both severe and common AD, T2DM, glaucoma, diabetes, and metabolic syndrome diseases. Clinical pharmacology research can support the administration of *E. rauwolffii* for pharmacological applications in people with the indicated disorders.

## Figures and Tables

**Table 1:** The extraction yield (%), total phenolics (mg GAE/g extract) and total flavonoids (mg QE/g extract) of *E. rauwolffii* (EEER: Ethanol extract of *E. rauwolffii* and WEER: Water extract of *E. rauwolffii*).

Extracts	Extraction Yield (%)	Phenolics (mg GAE/g Extract)	Flavonoids (mg QE/g Extract)
EEER	28.60	298.54 ± 0.01	178.95 ± 0.01
WEER	13.50	189.78 ± 0.01	89.37 ± 0.01

**Table 2:** Quantitative LC-MS/MS results of EEER (ethanol extract of *E. rauwolffii*) and WEER (water extract of *E. rauwolffii*) as mg/g extract.

No	Analyte	RT ^a^	M.I. (*m*/*z*) ^b^	F.I. (*m*/*z*) ^c^	EEER	WEER
1	Quinic acid	3.0	190.8	93.0	1.793	0.216
2	Fumaric acid	3.9	115.2	40.9	4.234	<LOD
3	Aconitic acid	4.0	172.8	129.0	0.073	<LOD
4	Gallic acid	4.4	168.8	79.0	^d^ <LOD	<LOD
5	Epigallocatechin	6.7	304.8	219.0	<LOD	<LOD
6	Protocatechuic acid	6.8	152.8	108.0	0.363	<LOD
7	Catechin	7.4	288.8	203.1	<LOD	<LOD
8	Gentisic acid	8.3	152.8	109.0	<LOD	<LOD
9	Chlorogenic acid	8.4	353.0	85.0	<LOD	<LOD
10	Protocatechuic aldehyde	8.5	137.2	92.0	0.074	<LOD
11	Tannic acid	9.2	182.8	78.0	<LOD	<LOD
12	Epigallocatechin gallate	9.4	457.0	305.1	<LOD	<LOD
13	Cynarine	9.8	515.0	191.0	<LOD	<LOD
14	4-OH-benzoic acid	10.5	137.2	65.0	<LOD	<LOD
15	Epicatechin	11.6	289.0	203.0	<LOD	<LOD
16	Vanillic acid	11.8	166.8	108.0	<LOD	<LOD
17	Caffeic acid	12.1	179.0	134.0	3.999	<LOD
18	Syringic acid	12.6	196.8	166.9	<LOD	<LOD
19	Vanillin	13.9	153.1	125.0	<LOD	<LOD
20	Syringic aldehyde	14.6	181.0	151.1	<LOD	<LOD
21	Daidzin	15.2	417.1	199.0	<LOD	<LOD
22	Epicatechin gallate	15.5	441.0	289.0	<LOD	<LOD
23	Piceid	17.2	391.0	135/106.9	<LOD	<LOD
24	p-Coumaric acid	17.8	163.0	93.0	4.996	0.218
25	Ferulic acid-D3-IS ^f^	18.8	196.2	152.1	N.A. ^e^	N.A.
26	Ferulic acid	18.8	192.8	149.0	<LOD	<LOD
27	Sinapic acid	18.9	222.8	193.0	<LOD	<LOD
28	Coumarin	20.9	146.9	103.1	<LOD	<LOD
29	Salicylic acid	21.8	137.2	65.0	0.208	0.018
30	Cynaroside	23.7	447.0	284.0	11.077	<LOD
31	Miquelianin	24.1	477.0	150.9	<LOD	<LOD
32	Rutin-D3-IS	25.5	612.2	304.1	N.A.	N.A.
33	Rutin	25.6	608.9	301.0	0.04	<LOD
34	Isoquercitrin	25.6	463.0	271.0	0.054	<LOD
35	Hesperidin	25.8	611.2	449.0	<LOD	<LOD
36	*o*-Coumaric acid	26.1	162.8	93.0	<LOD	<LOD
37	Genistin	26.3	431.0	239.0	<LOD	<LOD
38	Rosmarinic acid	26.6	359.0	197.0	<LOD	<LOD
39	Ellagic acid	27.6	301.0	284.0	<LOD	<LOD
40	Cosmosiin	28.2	431.0	269.0	4.003	<LOD
41	Quercitrin	29.8	447.0	301.0	N.D.	<LOD
42	Astragalin	30.4	447.0	255.0	0.061	<LOD
43	Nicotiflorin	30.6	592.9	255.0/284.0	0.058	<LOD
44	Fisetin	30.6	285.0	163.0	<LOD	<LOD
45	Daidzein	34.0	253.0	223.0	<LOD	<LOD
46	Quercetin-D3-IS	35.6	304.0	275.9	N.A.	N.A.
47	Quercetin	35.7	301.0	272.9	<LOD	<LOD
48	Naringenin	35.9	270.9	119.0	0.036	0.006
49	Hesperetin	36.7	301.0	136.0/286.0	<LOD	<LOD
50	Luteolin	36.7	284.8	151.0/175.0	0.412	0.033
51	Genistein	36.9	269.0	135.0	<LOD	<LOD
52	Kaempferol	37.9	285.0	239.0	<LOD	<LOD
53	Apigenin	38.2	268.8	151.0/149.0	0.109	0.201
54	Amentoflavone	39.7	537.0	417.0	<LOD	<LOD
55	Chrysin	40.5	252.8	145.0/119.0	<LOD	<LOD
56	Acacetin	40.7	283.0	239.0	<LOD	<LOD

^a^ RT: retention time, ^b^ M.I.: molecular ions, ^c^ F.I.: fragment ions, ^d^ <LOD.: limit of detection, ^e^ N.A.: not applicable, and ^f^ IS: internal standard.

**Table 3:** Cupric ions (Cu^2+^), ferric ions (Fe^3+^) and FRAP reduction assays of EEER (ethanol extract of *E. rauwolffii*) and WEER (water extract of *E. rauwolffii*).

Antioxidants	Cu^2+^ Reducing		FRAP Reducing		Fe^3+^ Reducing	
	λ_450_	r^2^	λ_593_	r^2^	λ_700_	r^2^
BHA	1.58 ± 0.02	0.9912	1.75 ± 0.04	0.9984	1.56 ± 0.03	0.9997
BHT	2.15 ± 0.07	0.9990	1.50 ± 0.08	0.9992	2.08 ± 0.06	0.9985
α-Tocopherol	1.47 ± 0.04	0.9992	-	-	0.47 ± 0.01	0.9705
Trolox	1.04 ± 0.10	0.9882	0.84 ± 0.01	0.9990	1.46 ± 0.01	0.9903
EEER	0.78 ± 0.46	0.9635	0.59 ± 0.01	0.9944	1.57 ± 0.04	0.9903
WEER	- *	- *	0.53 ± 0.01	0.9942	- *	- *

* It could not be determined despite several repetitions.

**Table 4:** Half maximal radical scavenging concentrations (IC_50_) of EEER and WEER towards DPPH and ABTS radicals (EEER: ethanol extract of *E. rauwolffii* and WEER: water extract of *E. rauwolffii*).

Antioxidants	DPPH^•^ Scavenging Ability		ABTS^•+^ Scavenging Ability	
	IC_50_	r^2^	IC_50_	r^2^
BHA	21.33 ± 5.46	0.9789	22.86 ± 5.97	0.9676
BHT	15.77 ± 3.13	0.9923	30.43 ± 5.53	0.9660
α-Tocopherol	56.88 ± 1.85	0.9877	26.87 ± 4.18	0.9809
Trolox	22.88 ± 9.17	0.9390	43.08 ± 2.26	0.9888
EEER	106.80 ± 1.88	0.9732	25.35 ± 1.42	0.9972
WEER	- *	- *	34.42 ± 1.69	0.9949

* It could not be determined despite several repetitions.

**Table 5:** Half maximal inhibition concentration (IC_50_, μg/mL) of *E. rauwolffii* extracts against α-glycosidase, acetylcholinesterase (AChE), butyrylcholinesterase (BChE), human carbonic anhydrase isoenzymes (hCAs I and II) (EEER: ethanol extract of *E. rauwolffii* and WEER: water extract of *E. rauwolffii*).

Samples	α-Glycosidase		AChE		BChE		hCA I		hCA II	
	IC_50_	r^2^	IC_50_	r^2^	IC_50_	r^2^	IC_50_	r^2^	IC_50_	r^2^
EEER	10.79 ± 5.61	0.9762	36.14 ± 4.61	0.9812	69.37 ± 7.36	0.9310	81.30 ± 5.95	0.9711	29.34 ± 1.38	0.9985
WEER	13.18 ± 5.77	0.9764	62.63 ± 1.67	0.9986	37.48 ± 0.27	0.9999	62.35 ± 8.03	0.9551	115.90 ± 3.3	0.9849
Standards *	9.43 ± 0.35	0.9995	8.82 ± 0.20	0.9836	15.51 ± 0.40	0.9836	15.83 ± 0.40	0.9836	9.96 ± 0.21	0.9930

* Acetazolamide was used as a standard inhibitor for hCA, Tacrine was used as a standard inhibitor for AChE, and Acarbose was used as a standard inhibitor for α-glycosidase, which was obtained from the literature.

## Data Availability

Data is publicly available in an accessible repository.

## Supplementary Materials

The following supporting information can be downloaded at: https://www.mdpi.com/article/10.3390/plants15091311/s1, Table S1: Validation Summart Report.
