# Supplementary material for: Antioxidant Properties and Enzyme Inhibitory Activities of Eminium rauwolffii: LC-MS/MS-Based Polyphenolic Profiling
Source: Plants (Basel). 2026 Apr 24;15(9):1311. doi: 10.3390/plants15091311 (PMC13165285; doi:10.3390/plants15091311)
Supplement: Supplementary file 1 [file plants-15-01311-s001.zip › plants-4246196-supplementary.pdf]

Supporting Information

Antioxidant Properties and Enzyme Inhibitory Activities of *Eminium rauwolffii*: LC-MS/MS-Based Polyphenolic Profiling

Kübra Aslan [1], Hasan Karageçili [2*], Veysel Tahiroğlu [3], Emrah Yerlikaya [4], Mustafa Abdullah Yılmaz [5], Mehmet Fidan [6] and İlhami Gülçin [1,7*]

[1]Department of Chemistry, Faculty of Science, Ataturk University, 25240-Erzurum, Türkiye;
kubra.aslan@atauni.edu.tr (K.A.); igulcin@atauni.edu.tr (I.G.)
[2]Department of Nursing, Faculty of Health Sciences, Siirt University, 56100-Siirt, Türkiye; hasankaragecili@siirt.edu.tr
[3]Faculty of Health Sciences, Department of Nursing, Sirnak University, Sirnak 73100, Türkiye; veysel.tahiroglu@sirnak.edu.tr
[4]Department of Nutrition and Dietetics, Faculty of Health Sciences, Siirt University, 56100-Siirt, Türkiye; emrahyerlikaya@siirt.edu.tr
[5]Faculty of Pharmacy, Department of Analytical Chemistry, Dicle University, 21280-Diyarbakır Türkiye; mustafaabdullahyilmaz@gmail.com
[6]Department of Biology, Faculty of Science and Arts, Siirt University, 56100-Siirt, Türkiye; mfidan7384@hotmail.com
[7]Rectorate of Agri Ibrahim Cecen University, 04100, Agri, Türkiye; igulcin@agri.edu.tr
* Correspondence: hasankaragecili@siirt.edu.tr; igulcin@atauni.edu.tr; Tel.: +90-4422314375

Table of Contents	Page
**Materials**	2
**Materials**	3
**Table S1.** Validation Summart Report	3
**Table S2.** Validation Summart Report (Continued)	4
**Table S3.** Validation Summart Report (Continued)	5
**Table S4.** Validation Summart Report (Continued)	6
**Table S5.** Validation Summart Report (Continued)	7
**Table S6.** Validation Summart Report (Continued)	9
**Table S7.** Validation Summart Report (Continued)	9
**References**	9

Materials

Reference phytochemical standards (quinic acid, gallic acid, fumaric acid, aconitic acid, protocatechuic acid, epigallocatechin, gentisic acid, catechin, chlorogenic acid, tannic acid, protocatechuic aldehyde, epigallocatechin gallate, epicatechin, 4-OH-benzoic acid, vanillic acid, syringic acid, caffeic acid, vanillin, daidzin, syringaldehyde, epicatechin gallate, ferulic acid, sinapic acid, p-coumaric acid, coumarin, cynaroside, salicylic acid, rutin, miquelianin, isoquercitrin, o-coumaric acid, hesperidin, genistin, ellagic acid, rosmarinic acid, cosmosiin, astragalin, quercitrin, nicotiflorin, daidzein, fisetin, quercetin, luteolin, naringenin, hesperetin, apigenin, genistein, kaempferol, chrysin, amentoflavone, and acacetin) were purchased from Sigma Aldrich (Steinheim, Germany). 1,5-Dicaffeoylquinic acid and the internal standards of rutin-D3, ferulic acid-D3, and quercetin-D3 were obtained from TRC (Toronto, Ontario, Canada).

Table S8. Validation Summart Report

No	Analyte	RT	Procursor ion	Product Ion	Ionization Mode	Calibration Equation	R^2	Interday RSD	Intraday RSD	Linaerity Range	LOD/LOQ	Intraday Recovery	Interday Recovery
1	Quinic acid	3.0	190.8	93.0	Neg	y=−0.0129989 + 2.97989×	0.996	0.69	0.51	0.1-5	25.7/33.3	1.0011	1.0083
2	Fumaric aid	3.9	115.2	40.9	Neg	y=−0.0817862 + 1.03467×	0.995	1.05	1.02	1-50	135.7/167.9	0.9963	1.0016
3	Aconitic acid	4.0	172.8	129.0	Neg	y=−0.7014530 + 32.9994×	0.971	2.07	0.93	0.1-5	16.4/31.4	0.9968	1.0068
4	Gallic acid	4.4	168.8	79.0	Neg	y = 0.0547697 + 20.8152×	0.999	1.60	0.81	0.1-5	13.2/17.0	1.0010	0.9947
5	Epigallocatechin	6.7	304.8	219.0	Neg	y=−0.00494986 +	0.998	1.22	0.73	1-50	237.5/265.9	0.9969	1.0040

						0.0483704×							
6	Protocatechuic acid	6.8	152.8	108.0	Neg	y = 0.211373 + 12.8622×	0.957	1.43	0.76	0.1-5	21.9/38.6	0.9972	1.0055
7	Catechin	7.4	288.8	203.1	Neg	y=−0.00370053 + 0.431369×	0.999	2.14	1.08	0.2-10	55.0/78.0	1.0024	1.0045
8	Gentisic acid	8.3	152.8	109.0	Neg	y=−0.0238983 + 12.1494×	0.997	1.81	1.22	0.1-5	18.5/28.2	0.9963	1.0077
9	Chlorogenic acid	8.4	353.0	85.0	Neg	y = 0.289983 + 36.3926×	0.995	2.15	1.52	0.1-5	13.1/17.6	1.0000	1.0023
**No**	**Analyte**	**RT**	**Procursor ion**	**Product Ion**	**Ionization Mode**	**Calibration Equation**	**R^2**	**Interday RSD**	**Intraday RSD**	**Linaerity Range**	**LOD/LOQ**	**Intraday Recovery**	**Interday Recovery**
10	Protocatechuic aldehyde	8.5	137.2	92.0	Neg	y = 0.257085 + 25.4657×	0.996	2.08	0.57	0.1-5	15.4/22.2	1.0002	0.9988
11	Tannic acid	9.2	182.8	78.0	Neg	y = 0.0126307 + 26.9263×	0.999	2.40	1.16	0.05-2.5	15.3/22.7	0.9970	0.9950
12	Epigallocatechin gallate	9.4	457.0	305.1	Neg	y=−0.0380744 + 1.61233×	0.999	1.30	0.63	0.2-10	61.0/86.0	0.9981	1.0079
13	1,5-dicaffeoylquinic acid	9.8	515.0	191.0	Neg	y=−0.0164044 + 16.6535×	0.999	2.42	1.48	0.1-5	5.8/9.4	0.9983	0.9997
14	4-OH Benzoic acid	10.5	137,2	65.0	Neg	y=−0.0240747 + 5.06492×	0.999	1.24	0.97	0.2-10	68.4/88.1	1.0032	1.0068

15	Epicatechin	11.6	289.0	203.0	Neg	y=−0.0172078 + 0.0833424×	0.996	1.47	0.62	1-50	139.6/161.6	1.0013	1.0012
16	Vanilic acid	11.8	166.8	108.0	Neg	y=−0.0480183 + 0.779564×	0.999	1.92	0.76	1-50	141.9/164.9	1.0022	0.9998
17	Caffeic acid	12.1	179.0	134.0	Neg	y = 0.120319 + 95.4610×	0.999	1.11	1.25	0.05-2.5	7.7/9.5	1.0015	1.0042
18	Syringic acid	12.6	196.8	166.9	Neg	y=−0.0458599 + 0.663948×	0.998	1.18	1.09	1-50	82.3/104.5	1.0006	1.0072
19	Vanillin	13.9	153.1	125.0	Poz	y = 0.00185898 + 20.7382×	0.996	1.10	0.85	0.1-5	24.5/30.4	1.0009	0.9967
**No**	**Analyte**	**RT**	**Procursor ion**	**Product Ion**	**Ionization Mode**	**Calibration Equation**	**R2**	**Interday RSD**	**Intraday RSD**	**Linaerity Range**	**LOD/LOQ**	**Intraday Recovery**	**Interday Recovery**
20	Syringic aldehyde	14.6	181.0	151.1	Neg	y=−0.0128684 + 7.90153×	0.999	2.51	0.77	0.4-20	19.7/28.0	1.0001	0.9964
21	Daidzin	15.2	417.1	199.0	Poz	y = 9.45747 + 152.338×	0.996	2.25	1.32	0.05-2.5	7.0/9.5	0.9955	1.0017
22	Epicatechin gallate	15.5	441.0	289.0	Neg	y=−0.0142216 + 1.06768×	0.997	1.63	1.28	0.1-5	19.5/28.5	0.9984	0.9946
23	Piceid	17.2	391.0	135/106.9	Poz	y = 0.00772525 + 25.4181×	0.999	1.94	1.16	0.05-2.5	13.8/17.8	1.0042	0.9979
24	p-Coumaric acid	17.8	163.0	93.0	Neg	y = 0.0249034 + 18.5180×	0.999	1.92	1.43	0.1-5	25.9/34.9	1.0049	1.0001

25	Ferulic acid-D3 ISh	18.8	196.2	152.1	Neg	N.A.	N.A.	N.A.	N.A.	N.A.	N.A.	N.A.	N.A.
26	Ferulic acid	18.8	192.8	149.0	Neg	y=−0.0735254 + 1.34476×	0.999	1.44	0.53	1-50	11.8/15.6	0.9951	0.9976
27	Sinapic acid	18.9	222.8	193.0	Neg	y=−0.0929932 + 0.836324×	0.999	1.45	0.52	0.2-10	65.2/82.3	1.0031	1.0037
28	Coumarin	20.9	146.9	103.1	Poz	y = 0.0633397 + 136.508×	0.999	2.11	1.54	0.05-2.5	214.2/247.3	0.9950	0.9958
**No**	**Analyte**	**RT**	**Procursor ion**	**Product Ion**	**Ionization Mode**	**Calibration Equation**	**R2**	**Interday RSD**	**Intraday RSD**	**Linaerity Range**	**LOD/LOQ**	**Intraday Recovery**	**Interday Recovery**
29	Salicylic acid	21.8	137.2	65.0	Neg	y = 0.239287 + 153.659×	0.999	1.48	1.18	0.05-2.5	6.0/8.3	0.9950	0.9998
30	Cynaroside	23.7	447.0	284.0	Neg	y = 0.280246 + 6.13360×	0.997	1.56	1.12	0.05-2.5	12.1/16.0	1.0072	1.0002
31	Miquelianin	24.1	477.0	150.9	Neg	y=-0.00991585 + 5.50334×	0.999	1.31	0.95	0.1-5	10.6/14.7	0.9934	0.9965
32	Rutin-D3-ISh	25.5	612.2	304.1	Neg	N.A.	N.A.	N.A.	N.A.	N.A.	N.A.	N.A.	N.A.
33	Rutin	25.6	608.9	301.0	Neg	y=-0.0771907 + 2.89868×	0.999	1.38	1.09	0.1-5	15.7/22.7	0.9977	1.0033
34	isoquercitrin	25.6	463.0	271.0	Neg	y=-0.111120 + 4.10546×	0.998	2.13	0.78	0.1-5	8.7/13.5	1.0057	0.9963
35	Hesperidin	25.8	611.2	449.0	Poz	y = 0.139055 +	0.999	1.84	1.35	0.1-5	19.0/26.0	0.9967	1.0043

						13.2785×							
36	o-Coumaric acid	26.1	162.8	93.0	Neg	y = 0.00837193 + 11.2147×	0.999	2.11	1.46	0.1-5	31.8/40.4	1.0044	0.9986
37	Genistin	26.3	431.0	239.0	Neg	y = 1.65808 + 7.57459×	0.991	2.01	1.28	0.1-5	14.9/21.7	1.0062	1.0047
38	Rosmarinic acid	26.6	359.0	197.0	Neg	y=-0.0117238 + 8.04377×	0.999	1.24	0.86	0.1-5	16.2/21.2	1.0056	1.0002
**No**	**Analyte**	**RT**	**Procursor ion**	**Product Ion**	**Ionization Mode**	**Calibration Equation**	**R2**	**Interday RSD**	**Intraday RSD**	**Linaerity Range**	**LOD/LOQ**	**Intraday Recovery**	**Interday Recovery**
39	Ellagic acid	27.6	301.0	284.0	Neg	y = 0.00877034 + 0.663741×	0.999	1.57	1.23	0.4-20	56.9/71.0	1.0005	1.0048
40	Cosmosiin	28.2	431.0	269.0	Neg	y=-0.708662 + 8.62498×	0.998	1.65	1.30	0.1-5	6.3/9.2	0.9940	0.9973
41	Quercitrin	29.8	447.0	301.0	Neg	y=-0.00153274 + 3.20368×	0.999	2.24	1.16	0.1-5	4.8/6.4	0.9960	0.9978
42	Astragalin	30.4	447.0	255.0	Neg	y = 0.00825333 + 3.51189×	0.999	2.08	1.72	0.1-5	6.6/8.2	0.9968	0.9957
43	Nicotiflorin	30.6	592.9	255.0/284.0	Neg	y = 0.00499333 + 2.62351×	0.999	1.48	1.23	0.05-2.5	11.9/16.7	0.9954	1.0044
44	Fisetin	30.6	285.0	163.0	Neg	y = 0.0365705 + 8.09472×	0.999	1.75	1.19	0.1-5	10.1/12.7	0.9980	1.0042
45	Daidzein	34.0	253.0	223.0	Neg	y=-0.0329252 +	0.999	2.18	1.73	0.1-5	9.8/11.6	0.9926	0.9963

						6.23004×							
46	Quercetin-D3-ISh	35.6	304.0	275.9	Neg	N.A.	N.A.	N.A.	N.A.	N.A.	N.A.	N.A.	N.A.
47	Quercetin	35.7	301.0	272.9	Neg	y=+0.00597342 + 3.39417×	0.999	1.89	1.38	0.1-5	15.5/19.0	0.9967	0.9971
48	Naringenin	35.9	270.9	119.0	Neg	y=-0.00393403 + 14.6424×	0.999	2.34	1.69	0.1-5	2.6/3.9	1.0062	1.0020
**No**	**Analyte**	**RT**	**Procursor ion**	**Product Ion**	**Ionization Mode**	**Calibration Equation**	**R2**	**Interday RSD**	**Intraday RSD**	**Linaerity Range**	**LOD/LOQ**	**Intraday Recovery**	**Interday Recovery**
49	Hesperetin	36.7	301.0	136.0/286.0	Neg	y=+0.0442350 + 6.07160×	0.999	2.47	2.13	0.1-5	7.1/9.1	0.9998	0.9963
50	Luteolin	36.7	284.8	151.0/175.0	Neg	y=-0.0541723 + 30.7422×	0.999	1.67	1.28	0.05-2.5	2.6/4.1	0.9952	1.0029
51	Genistein	36.9	269.0	135.0	Neg	y=-0.00507501 + 12.1933×	0.999	1.48	1.19	0.05-2.5	3.7/5.3	1.0069	1.0012
52	Kaempferol	37.9	285.0	239.0	Neg	y=-0.00459557 + 3.13754×	0.999	1.49	1.26	0.05-2.5	10.2/15.4	0.9992	0.9990
53	Apigenin	38.2	268.8	151.0/149.0	Neg	y = 0.119018 + 34.8730×	0.998	1.17	0.96	0.05-2.5	1.3/2.0	0.9985	1.0003
54	Amentoflavone	39.7	537.0	417.0	Neg	y = 0.727280 +	0.992	1.35	1.12	0.05-2.5	2.8/5.1	0.9991	1.0044

						33.3658×							
55	Chrysin	40.5	252.8	145.0/119.0	Neg	y=-0.0777300 + 18.8873×	0.999	1.46	1.21	0.05-2.5	1.5/2.8	0.9922	1.0050
56	Acacetin	40.7	283.0	239.0	Neg	y=-0.559818 + 163.062×	0.997	1.67	1.28	0.02-1	1.5/2.5	0.9949	1.0011

References

[1] M.A. Yılmaz (2020). Simultaneous quantitative screening of 53 phytochemicals in 33 species of medicinal and aromatic plants: A detailed, robust and comprehensive LC–MS/MS method validation. *Ind. Crops Prod.* **149**, 112347.
